# Supplementary figures and images for: Inflammatory markers in pregnancy – identifying drivers in four large cohorts
Source: Front Immunol. 2025 Jun 9;16:1561798. doi: 10.3389/fimmu.2025.1561798 (PMC12183797; doi:10.3389/fimmu.2025.1561798)

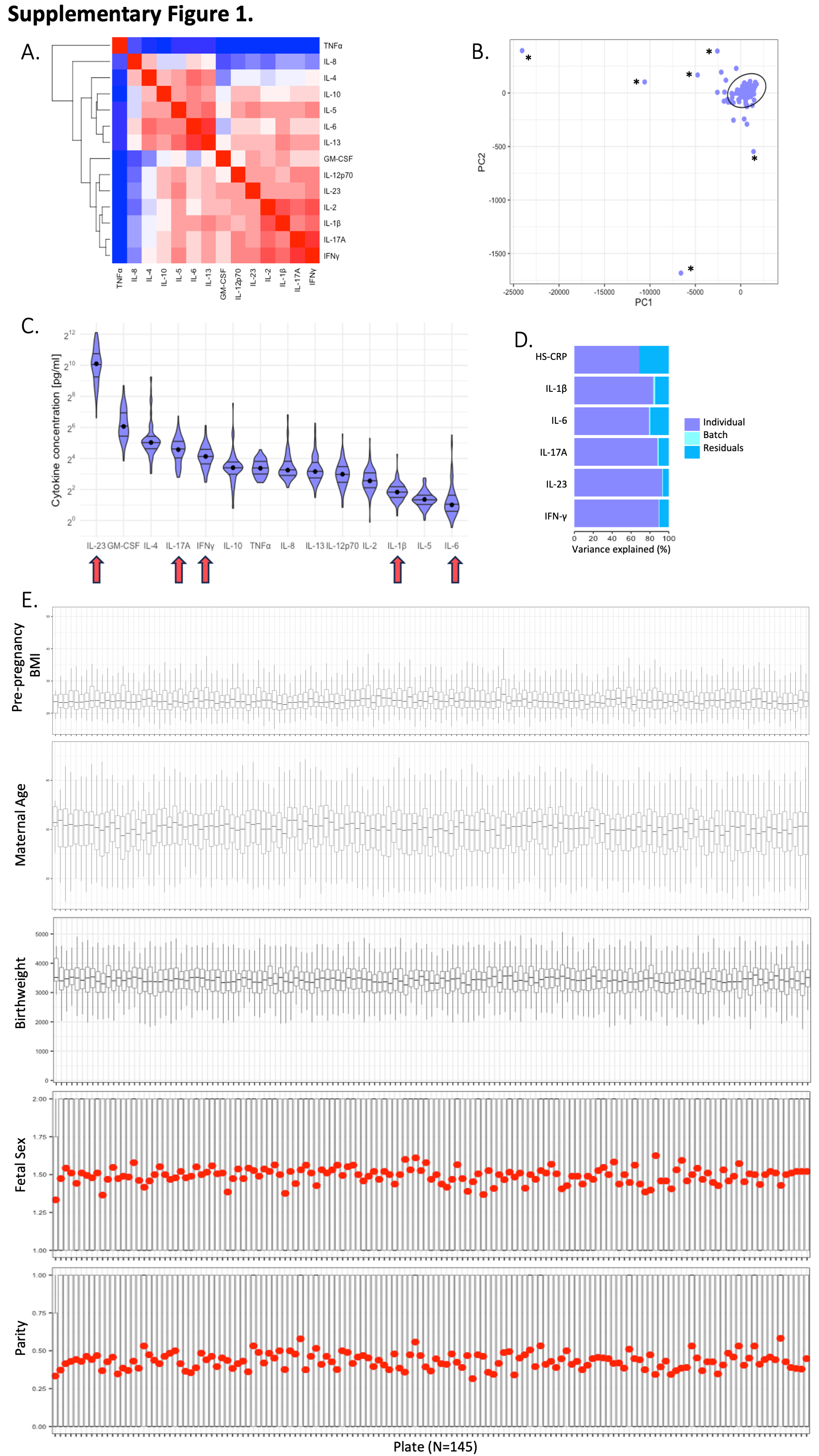

Supplement: Supplementary file 2 [file Image1.tiff]

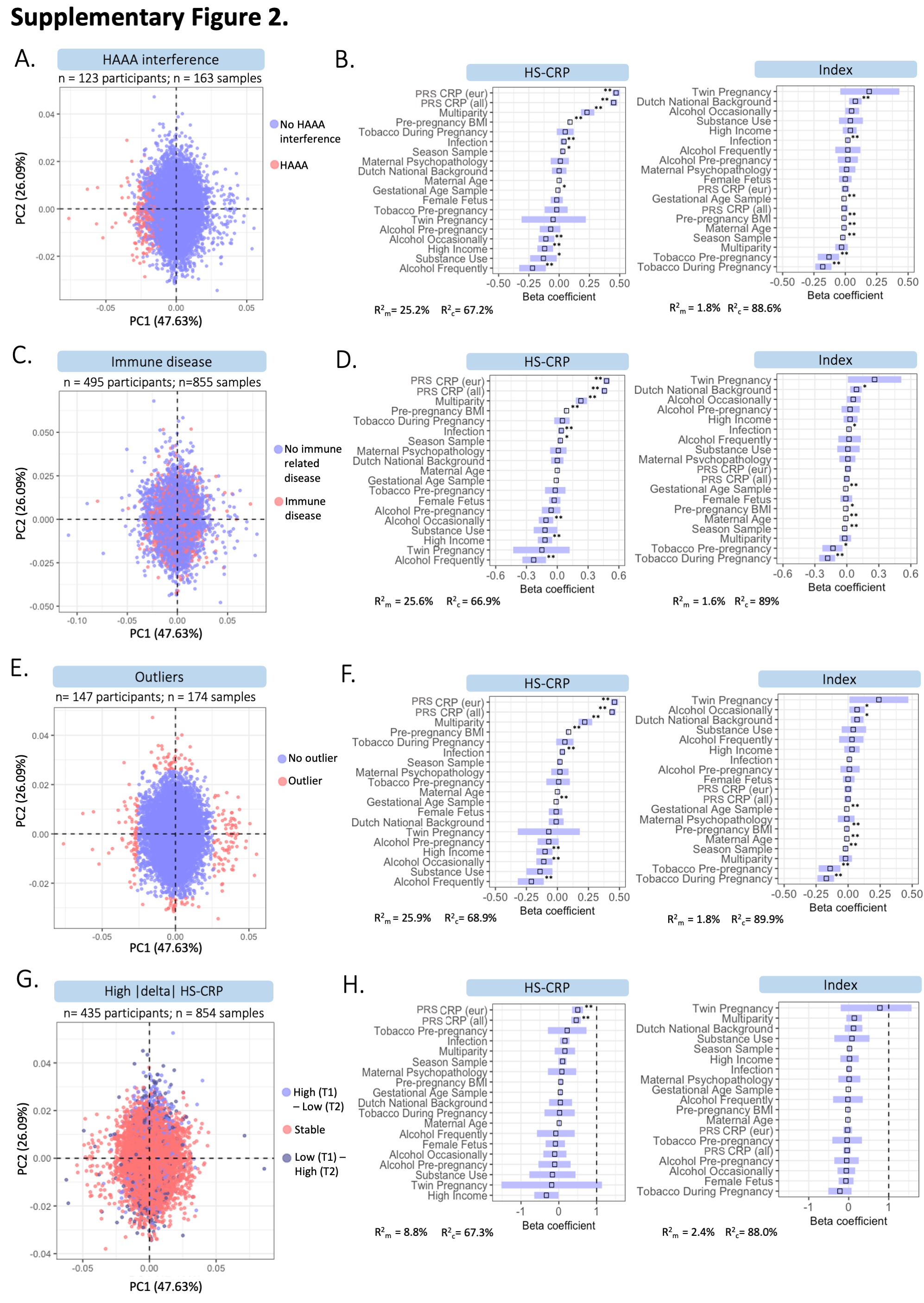

Supplement: Supplementary file 3 [file Image2.tiff]

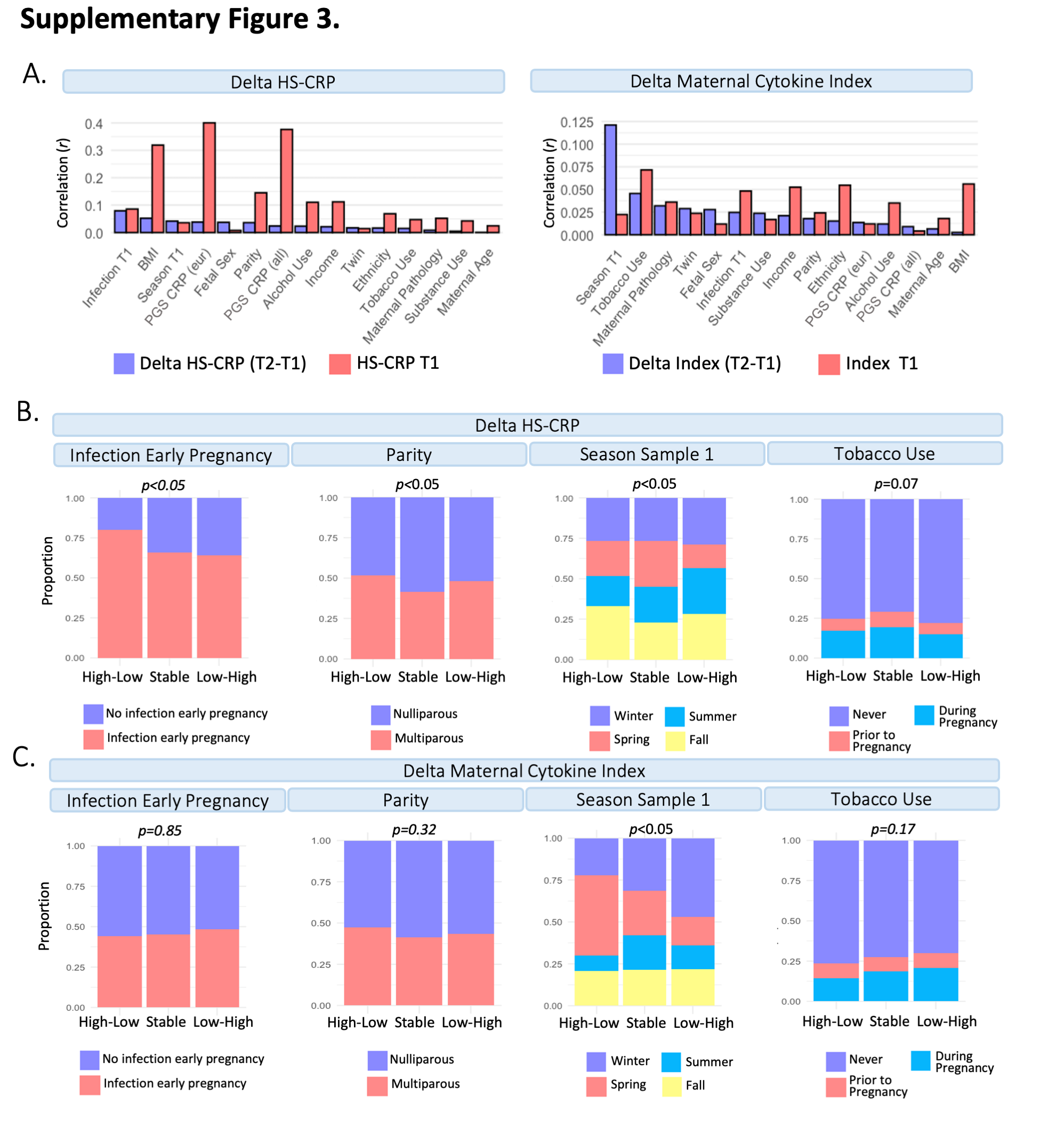

Supplement: Supplementary file 4 [file Image3.tiff]

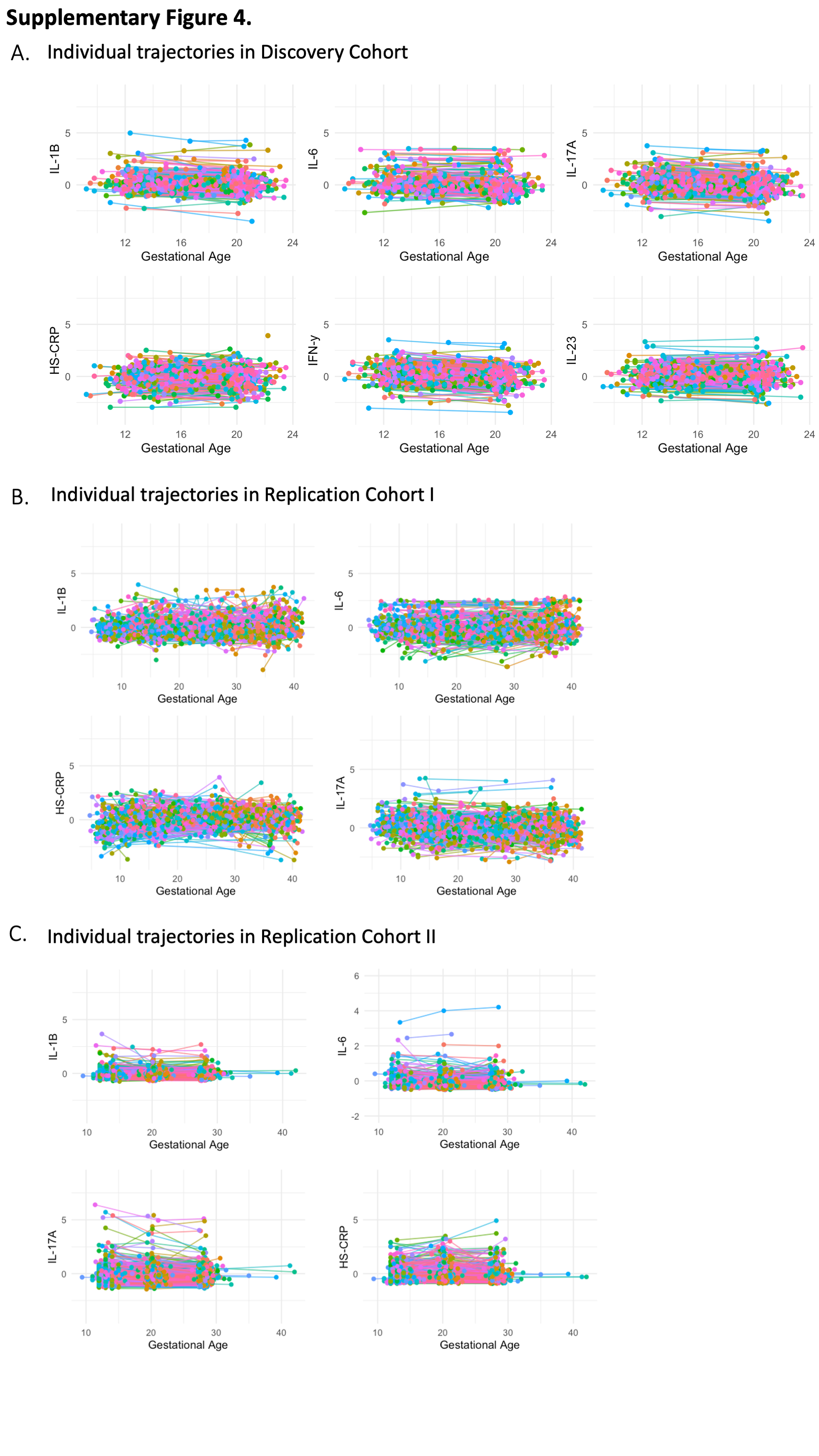

Supplement: Supplementary file 5 [file Image4.tiff]

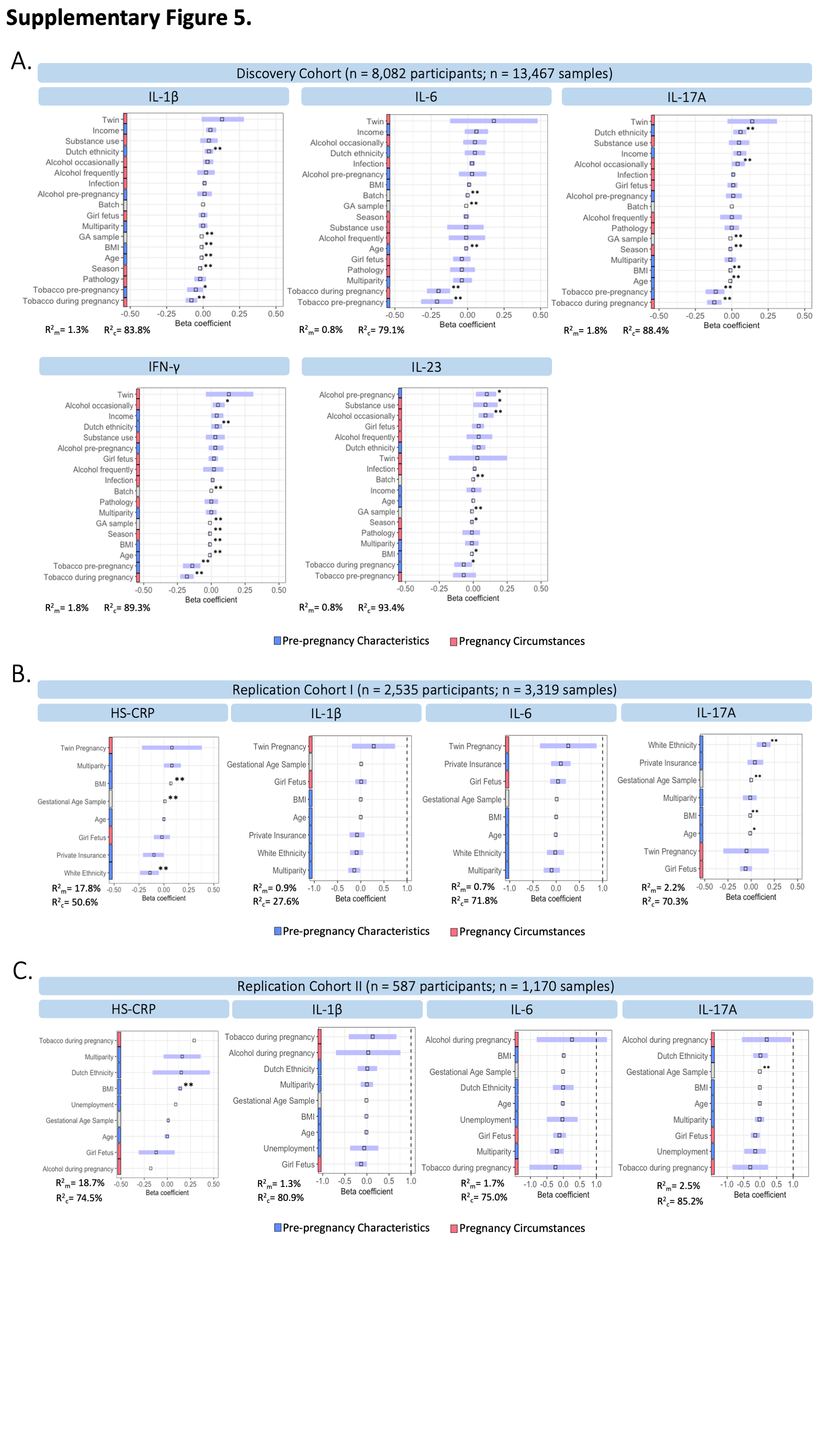

Supplement: Supplementary file 6 [file Image5.tiff]

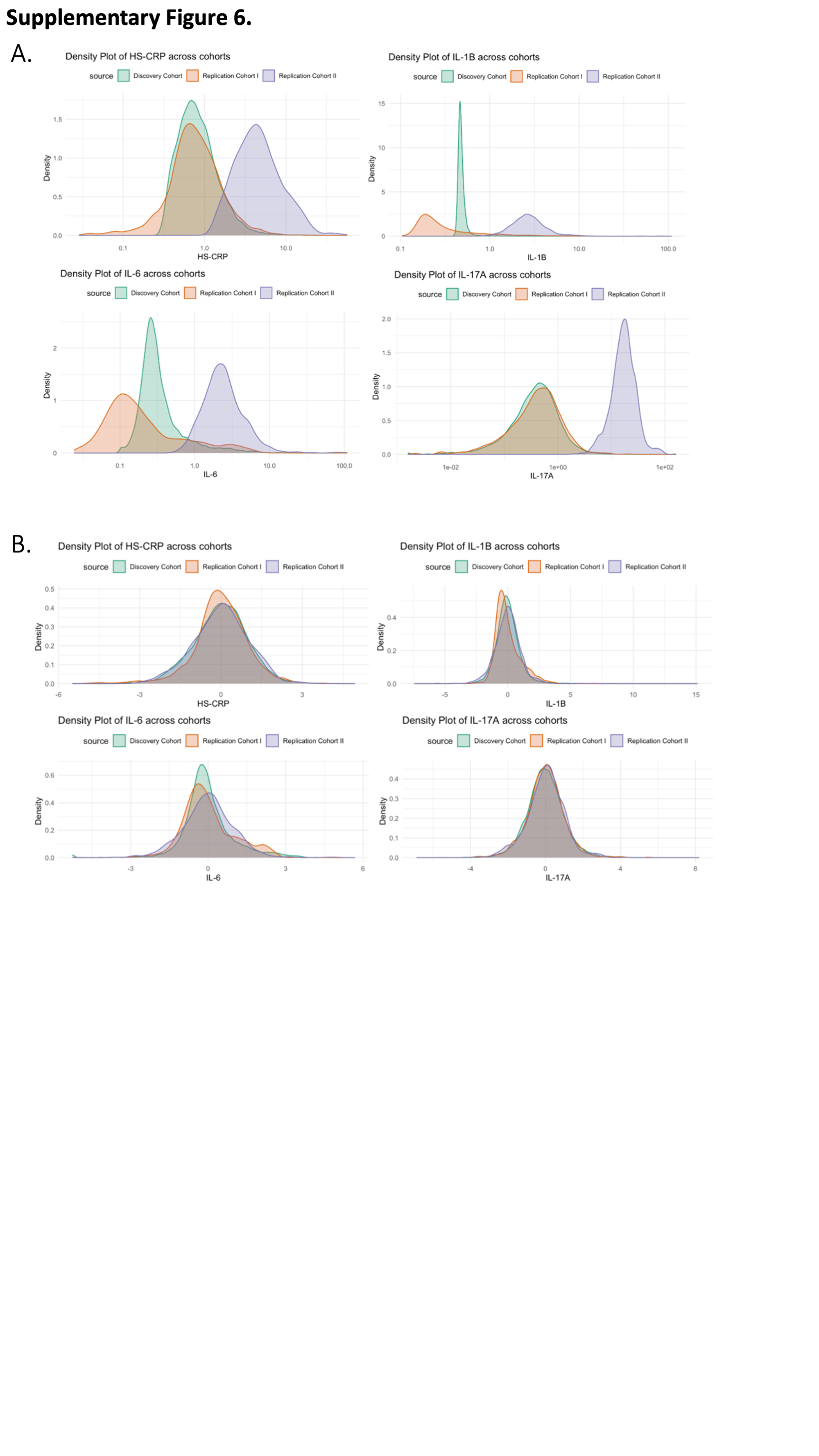

Supplement: Supplementary file 7 [file Image6.tiff]
